# Improved SNV Discovery in Barcode-Stratified scRNA-seq Alignments

**DOI:** 10.3390/genes12101558

**Published:** 2021-09-30

**Authors:** Prashant N. M., Hongyu Liu, Christian Dillard, Helen Ibeawuchi, Turkey Alsaeedy, Hang Chan, Anelia Dafinova Horvath

**Affiliations:** 1 McCormick Genomics and Proteomics Center, School of Medicine and Health Sciences, The George Washington University, Washington, DC 20037, USA; pnm27@gwmail.gwu.edu (P.N.M.); hliu5259@gwu.edu (H.L.); hibeawuchi@gwmail.gwu.edu (H.I.); turkey@gwmail.gwu.edu (T.A.); dcmkv2@gwmail.gwu.edu (H.C.); 2 Division of Animal Sciences, University of Missouri, Columbia, MO 65211, USA; 3 Department of Biochemistry and Molecular Medicine, School of Medicine and Health Sciences, The George Washington University, Washington, DC 20037, USA; cdillard49@gwmail.gwu.edu

**Keywords:** scRNA-seq, SNV, mutation, somatic mutation, SNP, expressed SNVs, SNV expression

## Abstract

Currently, the detection of single nucleotide variants (SNVs) from 10 x Genomics single-cell RNA sequencing data (scRNA-seq) is typically performed on the pooled sequencing reads across all cells in a sample. Here, we assess the gaining of information regarding SNV assessments from individual cell scRNA-seq data, wherein the alignments are split by cellular barcode prior to the variant call. We also reanalyze publicly available data on the MCF7 cell line during anticancer treatment. We assessed SNV calls by three variant callers—GATK, Strelka2, and Mutect2, in combination with a method for the cell-level tabulation of the sequencing read counts bearing variant alleles–SCReadCounts (single-cell read counts). Our analysis shows that variant calls on individual cell alignments identify at least a two-fold higher number of SNVs as compared to the pooled scRNA-seq; these SNVs are enriched in novel variants and in stop-codon and missense substitutions. Our study indicates an immense potential of SNV calls from individual cell scRNA-seq data and emphasizes the need for cell-level variant detection approaches and tools, which can contribute to the understanding of the cellular heterogeneity and the relationships to phenotypes, and help elucidate somatic mutation evolution and functionality.

## 1. Introduction

In single-cell studies, single nucleotide variant (SNV) analysis is an emerging and promising strategy to connect cell-level genetic variation to phenotypes and to interrogate the lineage relationships in heterogeneous cell populations. To detect single-cell SNVs from DNA, genome and exome sequencing experiments can be performed [1,2,3,4,5]. These studies have revealed enormous amounts of knowledge on cell-level genetic heterogeneity; however, they face challenges related to sample availability, unequal coverage, and amplification bias, and are relatively costly for large-scale applications. Recently, SNV assessments from single-cell RNA sequencing (scRNA-seq) experiments have started to emerge [6,7,8,9]. These analyses can complement DNA-based SNV-studies and maximize the potential of scRNA-seq datasets. Importantly, SNVs from scRNA-seq studies can provide crucial information on SNV functionality through studying allele-specific dynamics and their correlation to phenotype features, such as gene expression and splicing [10,11,12]. 

Among the scRNA-seq platforms, droplet-based technologies, such as 10x Genomics Chromium Single Cell 3′ and 5′ Expression workflows, are quickly gaining popularity. Presently, SNV detection from 10x scRNA-seq data is typically performed on the pooled sequencing reads (pseudo-bulk), where it utilizes those approaches optimized for bulk DNA- and RNA-variant calling [7,9,10,13]. These approaches often estimate quality control metrics, such as variant allele fraction (VAF) and/or genotype confidence, based on all sequencing reads in a sample [14,15]. As a result, SNVs with low VAF and/or uncertain genotypes in the pooled data are frequently filtered out. While it is widely acknowledged that post-zygotically occurring SNVs (such as somatic and mosaic mutations), being present in only a proportion of cells, can result in low VAF and uncertain genotypes, distinguishing those mutations from noise is difficult. Current approaches target somatic mutations by adjusting the thresholds for VAF- and genotype-based filtering and accounting for population SNV frequencies [16]. Nevertheless, without cell-level information, the detection of low-frequency SNVs is challenging. More recently, methods for barcode-aware SNV assessments have started to emerge [17,18], and pioneering studies have demonstrated strong advantages to such estimations in human scRNA-seq datasets. For example, barcode-aware SNV assessments in the setting of acute myeloid leukemia (AML) were used to define cells expressing specific somatic mutations that served as markers to distinguish clusters of putative AML cells from different types of normal cells [9]. 

In this study, we systematically assess the gain of information of SNV detection at the individual cell level, where the alignments are split by barcode prior to the variant call. We reasoned that such a setting enables VAF and genotype assessments per cell (as opposed to per sample), and is likely to result in retaining additional high-quality SNVs by variant callers. We performed our assessments using publicly available scRNA-seq data from the MCF7 cell line, coupled with matched whole-genome and targeted exome sequencing; all the sequencing data was previously generated as a part of a separate study [19].

## 2. Methods

### 2.1. Sequencing Datasets

To compare SNV assessments from single cells to those from pooled and bulk datasets, we utilized matched genome, exome, and scRNA-seq data from four time-points during bortezomib (Selleckchem, S1013, Texas, TX, USA) treatment of MCF7 cell line. The experimental design and the data generation were part of a separate study with a different scope, as reported by Ben-David et al [19]. MCF7 cell culturing and treatment are described in detail in the original study [19]. The sequencing datasets were downloaded from the NCBI Sequence Read Archive (SRA) (accessed on 1 April 2021) under the accession numbers SRR5945460 (MCF7, targeted exome), SRR5945478 (MCF7, whole genome), SRR10018149, SRR10018150, SRR10018151, and SRR10018152 (MCF7 before treatment (t0), after 12 h of exposure (t12), after 48 h of exposure (t48) or after 72 h of exposure, followed by a drug wash and 24 h of recovery (t96), respectively). The protocol followed 10x Genomics Chromium Single Cell 3′ Workflow, and the libraries were sequenced on an Illumina NextSeq 500 platform [19]. 

### 2.2. Data Processing: Alignment, Processing, Generation of Individual scRNA-seq Alignments

The targeted exome and the whole genome sequencing reads were aligned to the latest version of the human genome reference (GRCh38, Dec 2013) using BWA v.0.7.17 default settings [20]. The pooled sequencing reads from the scRNA-seq datasets were aligned using the STARsolo module of STAR v.2.7.7a in 2-pass mode, with transcript annotations from the assembly GRCh38.79 [12,21]. To generate individual cell alignments we adopted a publicly available python script that splits the pooled scRNA-seq alignments, based on cellular barcode [22]. 

### 2.3. Variant Call

For all DNA and RNA datasets, a variant call was performed, applying the HaplotypeCaller module of GATK v.4.2.0.0, in parallel with Strelka2 v.2.9.10; both tools were used under their default settings [14,15]. For RNA datasets, the HaplotypeCaller was preceded by the assignment of read groups using the GATK module AddOrReplaceReadGroups, followed by splitting reads that contain Ns in their cigar string with the GATK module SplitNCigarReads. For the initial comparisons that included the DNA datasets, no filtering was applied on the SNV calls from the pooled or bulked variant calls. The SNV calls from the individual alignments were filtered using bcftools v.1.10.2 [23] according to the following criteria: QUAL (Phred-scaled probability)  >  100, MQ (mapping quality)  >  60, and QD (quality by depth) >  2. The same filtering was applied on the SNV calls from pooled alignments for the analyses of distribution on novel SNVs and functional annotations. SNV loci were annotated using SeattleSeq v.16.00 (dbSNP build 154), and those SNV loci positioned in repetitive regions were removed. Thus, processed SNV calls were subject to the above-described analyses.

### 2.4. Gene Expression Estimation from scRNA-seq Data

To estimate gene expression, we used read-count matrices with the row gene counts per cell generated by STARsolo. We normalized and scaled the expression data using the SCTransform function, as implemented in Seurat v.3.0 [24,25]. The cell-feature distributions were then plotted to identify and filter out the outliers and low-quality cells, which we defined after examination of the cell feature distribution (Supplementary Figure S1). Specifically, based on the cell and feature distribution, we have filtered out: (1) cells with mitochondrial gene expression of between 7.5% and 15%, (2) cells with fewer than 1000 genes, and (3) cells with more than between 4500 and 5500 detected genes (to remove potential doublets). The Seurat-processed gene expression values were also used to remove batch effects and cell cycle effects (Supplementary Figure S2), as well as for cell type assessments and correlations with the expression of their harboring gene (cis-single-cell RNA eQTLs (cis-scReQTLs), see below). 

### 2.5. Cell Type Assessments

To define the similarity of the MCF7 clusters with known cell types, we used SingleR v.1.0.5 [26], as previously described [11]. Briefly, SingleR defines likely cell types, comparing the global expression profile of each cell to a large database of reference cells’ whole transcriptome expression (BluePrint + ENCODE datasets). To select the expression profile that is most similar to the tested cells, the analysis is rerun iteratively with the top cell types from the previous step. The tested cells may not represent 100% identity with the most similar reference pure cell types. Comparing our datasets against 259 bulk RNAseq profiles representing 24 main cell types and 43 subtypes, SingleR identified the highest correlations between the MCF7 derived subtypes and the following reference cell types: CD4+ T-cells, epithelial cells, macrophages, endothelial cells, erythrocytes, keratinocytes, plasma cells, and mesanglial cells (Supplementary Figure S3). 

### 2.6. VAF_RNA_ Estimation

Single-cell level VAF_RNA_ was assessed from the pooled scRNA-seq alignments using scReadCounts v.1.1.4, as we have previously described [18]. Briefly, when provided with barcoded scRNA-seq alignments and genomic loci of interest (with alleles), SCReadCounts tabulates the reference and variant read counts (n_ref_ and n_var_, respectively), and generates a cell-SNV matrix with the VAF_RNA_ estimated at a user-defined threshold of the minimum number of required sequencing reads (minR) for a confident VAF_RNA_ assessment. For the analysis presented herein, we used minR ≥ 3, which excludes from the analysis those positions covered by an insufficient number of reads (in this case 3). 

### 2.7. Correlation between VAF_RNA_ and Gene Expression

For each sceSNV called in more than 5 cells, we performed an analysis for a correlation between the VAF_RNA_ and the gene expression (cis-scReQTL) of the harboring gene, using scReQTL as previously described [11]. Briefly, the VAF_RNA_ estimates were correlated to the normalized gene expression values of the most variable genes, using a linear regression model as implemented in Matrix eQTL [27]. The top 15 principal components of gene expression were used as covariates. Cis-correlations were annotated as previously described for the bulk ReQTLs [28]. 

### 2.8. Statistical Analyses

Throughout the analysis, we used the default statistical tests (with built-in multiple testing corrections) implemented in the utilized software packages (Seurat, SingleR, Matrix eQTL, http://www.bios.unc.edu/research/genomic_software/Matrix_eQTL/, accessed on 5 July 2021), where a *p*-value of 0.05 was considered significant unless otherwise stated. For the estimation of significant scReQTL, we applied FDR as implemented in the Matrix eQTL package [27,29]. 

## 3. Results

### 3.1. Analytical Pipeline

To compare SNV assessments from single cells to those from pooled and bulk datasets, we utilized the matched genome, exome, and scRNA-seq data from multiple time-points during anticancer treatment (with bortezomib) of the human breast cancer cell line MCF7; the data was previously generated as a part of a separate study (reported by Ben-David et al., [19]) and publicly available. Specifically, scRNA-seq MCF7 was generated at four different time-points during bortezomib treatment: before treatment (t0) and after 12 h (t12), 48 h (t48), and 72  h of exposure, followed by a drug wash and 24  h of recovery (t96) [19], and accompanied by matched whole-genome sequencing (WGS) and deep (approximately 250× coverage) targeted exon sequencing (TES). TES targeted 334 genes that are commonly mutated in cancer (Profile OncoPanel v.3). We reasoned that the described data collection maximizes the identifiable SNVs across compatible DNA/RNA regions in bulk/pooled data. 

**Figure 1:**
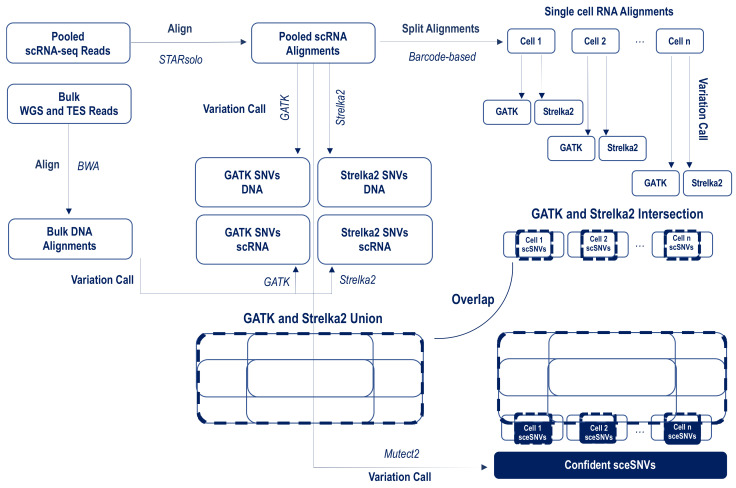
Analytical workflow for the identification of confident SNVs calls exclusively in the individual scRNA-seq alignments. The raw sequencing reads were aligned to GRCh38, using BWA for the DNA and STARsolo for the RNA data. GATK and Strelka were applied in parallel on both the pooled and individual scRNA-seq alignments. For the pooled/bulk data, all SNVs called by either GATK or Strelka2 SNVs were retained; for the individual alignments, the SNVs that were called confidently by both GATK and Strelka2 in each cell were retained. Single-cell exclusive SNVs (sceSNVs) were then outlined via overlapping the union of GATK and Strelka2 calls from the pooled/bulk scRNA and DNA, and the intersection of the GATK and Strelka2 calls from each individual alignment. To assess what percentage of sceSNVs are identifiable with callers specifically targeting SNVs in a low proportion of cells, we applied Mutect2 on the pooled alignments.

The analytical pipeline is presented in Figure 1. Our general strategy was to apply variant calling in parallel on the pooled and individual scRNA-seq alignments, in a setting that favors variant identification in the pooled data (relaxed or no filtering) over the individual (stringent quality filtering). We used three popular callers-GATK, Strelka2, and Mutect2, which have repeatedly demonstrated high-quality performance across both DNA and RNA sequencing data, including scRNA-seq data [6,7,14,15,16,30]. To retain the maximum number of identifiable SNVs in the pooled data, we applied GATK and Strelka2 in parallel, and then generated the union of the SNVs across WES, TES, and each of the 4 corresponding scRNA-seq datasets. To retain SNV calls with low VAF_RNA_, the variant calls were not filtered for the depth of allele coverage or confidence of the genotype call. 

For the individual cell alignments, we also applied GATK and Strelka2 in parallel. However, in contrast to the pooled data, where we aimed at maximizing the SNVs detection, here we aimed to outline the highest-confidence SNVs. Accordingly, the individual cell variant lists were filtered to retain only high-quality calls (Methods), and then, for each cell, the intersection between GATK and Strelka2 was generated for downstream analysis. We then asked if additional SNVs can be identified from the single-cell scRNA-seq alignments; we refer to these as single-cell exclusive SNVs, or sceSNVs. SceSNVs were defined as those called confidently by both GATK and Strelka in their individual cell alignments, and not called in any of the matched pooled/bulk scRNA or DNA datasets. Finally, to assess what percentage of sceSNVs are identifiable with callers specifically targeting SNVs in a low proportion of cells, we applied Mutect2 on the pooled alignments [16].

### 3.2. SNV Calls across TES, WGS, and scRNA-seq 

**Figure 2:**
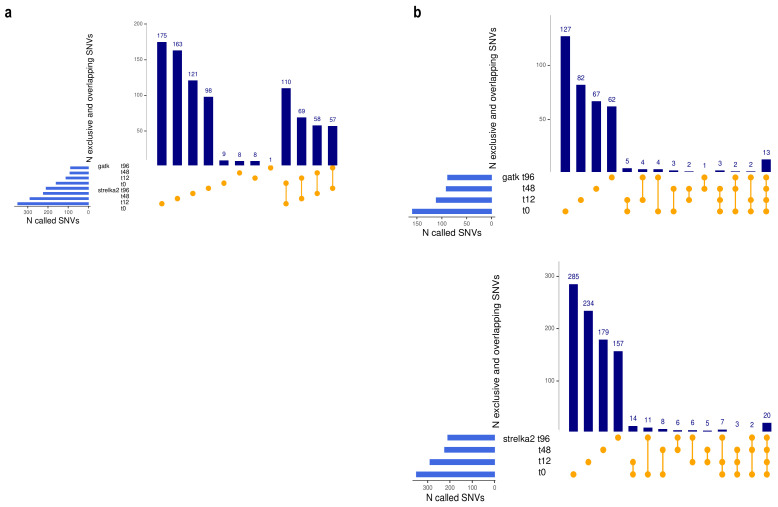
(**a**). Concordance between GATK and Strelka2 in variant calling from individual cell alignments. A higher number of SNVs were called by Strelka2, which also identifies the vast majority of the GATK calls. Note that the UpSet plots show the first 12 of all possible overlaps. (**b**). Shared and exclusive sceSNVs called by GATK (top) and Strelka2 (bottom) from scRNA-seq data generated at four time-points during drug treatment, showing the low overlap indicative of de novo SNVs.

For this analysis, we applied the above-described pipeline on the genomic regions compatible across TES, WGS, and scRNA-seq, which comprised the exons of the genes targeted by the POP exome capture. The numbers of common and exclusive SNVs in TES, WGS, and pooled and individual scRNA-seq alignments are shown in Table 1 and Supplementary Figure S4. In the individual alignments, Strelka2 called a 2- to 3-fold higher number of SNVs, which included the vast majority of the GATK calls (Figure 2a). Note that SNVs found exclusively in TES contain variants positioned in genes that are not expressed or are expressed at low levels in the studied sample, and are therefore not captured by RNA-sequencing.

Across the four scRNA-seq datasets, in the exonic regions of the 334 genes from the POP panel, the above pipeline identified between 38 and 73 sceSNVs (Supplementary Table S1; all of the sceSNV alignments were visually examined and the confidence of the call verified through the Integrative Genome Viewer (IGV). These numbers represented 48% and above of all confident individual alignment SNVs. In the pooled data, Mutect2 identified up to 11% of the sceSNVs. Thus, our analysis shows that even in settings strongly favoring variant discovery from bulk/pooled data, assessments of barcode-stratified individual cell alignments detect a substantially higher number of SNVs. 

We next assessed the proportion of SNVs shared across the four time-points post-drug treatment. This analysis was performed separately for the GATK and Strelka2, which showed highly concordant results. As seen in Figure 2b, sceSNVs show low overlap across the samples collected, over the four time-points of the drug treatment. This suggests enrichment with de novo arising SNVs, which is consistent with the finding of the original study on rapid MCF7 evolution during anticancer treatment [19].

### 3.3. Transcriptome-Wide SNVs Called Exclusively in the Individual Alignments

Following the above-described strategy, we next analyzed the transcriptome-wise shared and exclusive SNVs between the pooled and scRNA-seq alignments of the 4 time-points; the results are summarized in Table 2. Specifically, in the individual alignments, after stringent filtering of both GATK and Strelka2 calls, and retaining only the intersection of the two callers, we identified between 7000 and 14,000 SNVs per dataset that were not captured in the pooled scRNA-seq data by either GATK or Strelka2 (Supplementary Table S2). Of those, only up to 10% were identified using Mutect2. This observation aligns with the findings across the WGS/TES/RNA datasets on the exons of POP capture and suggests that transcriptome-wise application of variation call on barcode-stratified individual scRNA-seq data can identify thousands of SNVs in addition to those identified in the pooled scRNA-seq data.

We next assessed the number of cells bearing each of the sceSNVs. As expected, the maximum percentage of cells with sceSNVs represented up to 5% of the cells in the dataset (see Table 2), with the majority of the sceSNVs seen in only one cell (Supplementary Figure S5). We note that the high number of sceSNVs in only one cell is expected, given the fast genetic evolution of the studied system [19]. Between 318 and 636 sceSNVs (between 4 and 5% of all sceSNVs per sample) were called in two or more cells (see Supplementary Figure S5).

### 3.4. Novel and Known SNVs in the Individual scRNA-seq Alignments

We next analyzed the proportion of novel (previously undescribed) sceSNVs, and compared them to the proportion of novel SNVs identified in the pooled scRNA-seq datasets (pSNVs). For this analysis, we used pSNV calls that had been processed in the same way as the sceSNVs (i.e., the intersection of filtered GATK and Strelka2 calls). We defined as novel those SNVs not present in the NCBI Single Nucleotide Polymorphism database (DbSNP), the Catalog of Somatic Mutations in Cancer (COSMIC), or the ATLAS of RNA-editing events in humans (REDIportal) [31,32]. 

**Figure 3:**
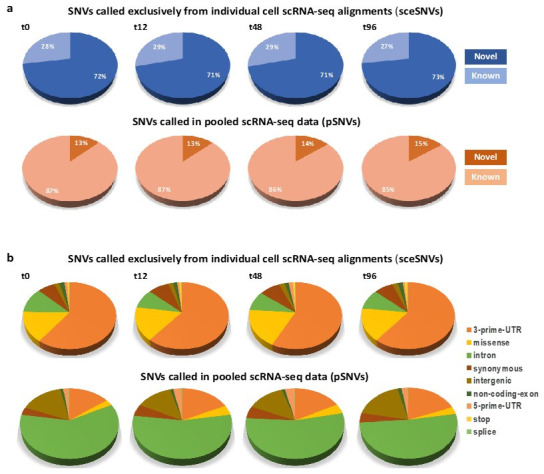
(**a**). Percentage of novel and known SNVs called exclusively in the individual alignments (sceSNVs, top) and in the pooled scRNA-seq data (pSNVs, bottom). An approximately 5-fold higher percentage of novel SNVs was seen in the individual cell alignments. (**b**). Distribution of functional annotations among the SNVs called exclusively in the individual alignments (top), as compared to the pooled scRNA-seq data (bottom). Significantly higher proportions of 3’-prime-UTR, missense and stop-codon SNVs were called in the individual alignments.

Notably, among the sceSNVs, we estimated a several-fold higher proportion of novel SNVs. Specifically, over 70% of the sceSNVs in each of the datasets were novel, compared to up to 15% of novel pSNVs called in the corresponding pooled scRNA-seq datasets (Figure 3a). This difference is likely due to the suggested high rate of de novo acquired mutations, present in a small proportion of cells and therefore detectable exclusively in the individual scRNA-seq alignments.

Next, we compared the distribution of predicted SNV functional annotations. This analysis revealed significant differences in the proportions of all the functional annotations between the sceSNVs and those SNVs called in the pooled scRNA-seq (Figure 3b). The largest annotation category for the sceSNVs was 3’-UTR, whereas, for the SNVs in the pooled data, it was intronic. SceSNVs also had a significantly higher proportion of coding variants, including stop-codon and missense substitutions (Table 3). The most striking difference was estimated for the stop-codon mutations, which showed an approximately 50-fold higher rate among the sceSNVs (around 1%, as opposed to up to 0.02% in the pooled SNVs). The missense substitutions had a 4- to 6-fold higher rate among the sceSNVs. In contrast, synonymous SNVs and SNVs in non-coding exons showed only up to a 2-fold higher rate in the sceSNVs. 

The observed differences in the functional categories in the sceSNVs require further attention and analyses on a higher number of samples. Like the high proportion of novel mutations, it is likely to be related to de novo sceSNVs, where different rates of mutation generation, mismatch repair, and purifying selections across different functional genomic regions play a role. Nevertheless, our observation highlights the potential of the scRNA-seq analyses to study mutation dynamics and evolution.

### 3.5. SceSNVs Expression

**Figure 4:**
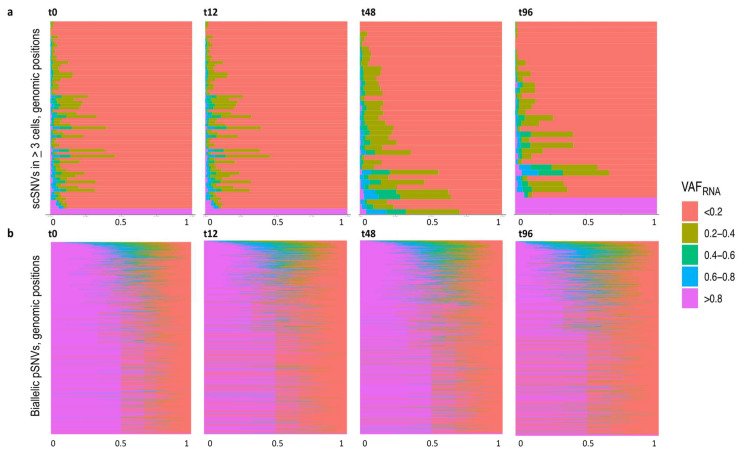
(**a**). ScVAF_RNA_ estimated at positions covered by a minimum of 3 sequencing reads (minR = 3) for sceSNVs called in 3 and more cells per dataset (y-axis). The majority of the positions have a VAF_RNA_ up to 0.2. Note that the plot is inclusive for all the cells with minR = 3 in the corresponding position, including those covered with reference reads only. The percentage of cells with a corresponding VAF_RNA_ is displayed on the x-axis. (**b**). ScVAF_RNA_ estimated at those positions covered by a minimum of 3 sequencing reads for biallelic pSNVs (y-axis). For most of the pSNVs, the VAF_RNA_ distribution is centered around 0.5, which is expected for germline heterozygous SNVs not subjected to monoallelic expression.

To estimate the expression of the sceSNVs, we applied SCReadCounts, as previously described [18]. For each cell, SCReadCounts tabulates the reference and variant counts of sequencing reads (n_ref_ and n_var_, respectively) for genomic positions of interest, and computes the expressed VAF (VAF_RNA_ = n_var_/(n_var_ + n_ref_)) at the desired depth threshold (minimum number of reads covering the position, minR). For this particular analysis, we estimated VAF_RNA_ at minR = 3. The distribution of VAF_RNA_ for sceSNVs called in 3 and more cells per dataset, and for all cells with 3 and more reads at the corresponding position, is shown in Figure 4a. The majority of the sceSNV positions showed VAF_RNA_ up to 0.2 across most of the cells. Note that this assessment includes also those cells with the only reference reads at the SNV position (i.e., VAF_RNA_ = 0). Such a VAF_RNA_ distribution is expected for those SNVs present in a small proportion of cells (i.e., de novo SNVs). In contrast, biallelic pSNVs show a VAF_RNA_ distribution centered around 0.5, which is generally expected for the majority of the heterozygous germline SNVs (Figure 4b).

**Figure 5:**
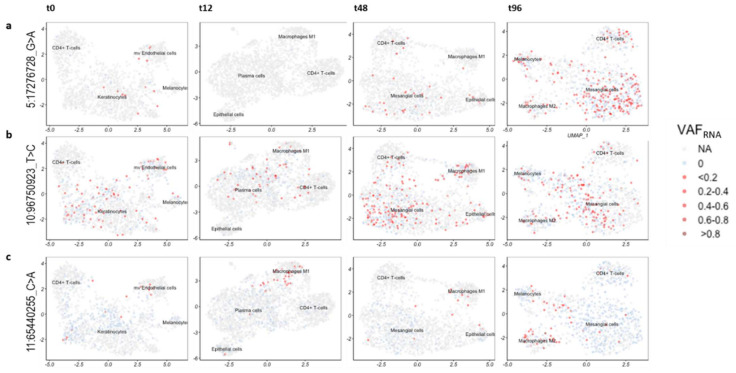
Two-dimensional UMAP projections with quantitative visualization (red) of sceSNVs VAF_RNA_. The light blue color indicates that the position is covered by at least 3 unique sequencing reads bearing the reference nucleotide, thereby signifying non-0 expression at the position. (**a**). SNV rs1161976348 (5:17276721_G > A) in the 3’-UTR of the gene *BASP1*. A higher proportion of cells appear to express the SNV at later time-points post-anti-cancer treatment, especially at t96. (**b**). Novel intergenic SNV (10:96750923_T > C) showing a relatively even distribution across the different cell types and clusters of the 4 post-treatment time-points. (**c**). Novel SNV positioned at 11:65440255 (C > A) in a non-coding exon of the gene *NEAT1*, expressed preferentially in the microphages.

To explore if cells bearing certain sceSNVs have related gene expression features, we assessed the sceSNV expression in the individual cells after graph-based cell clustering. For this analysis, we processed the scRNA-seq datasets as we have previously described [11,18]. Briefly, after alignment with STARsolo [12] and quality filtering, the gene-expression matrices were processed using Seurat [25] to normalize gene expression, and corrected for batch- and cell-cycle effects. The normalized gene expression values were then used to assign likely cell types using SingleR and to provide context for cells carrying particular SNVs [26] (Methods). We then visualized VAF_RNA_ in the cells bearing sceSNVs over the UMAP two-dimensional projections of the scRNA-seq datasets; examples are shown in Figure 5.

Some sceSNVs showed different expression across the four treatment time-points. One example is rs1161976348 (5:17276721_G > A in the 3’-UTR of the gene *BASP1*), which appeared to be expressed in a higher proportion of cells at later time-points, and especially at t96 (Figure 5a). Other sceSNVs (such as the novel intergenic SNV 10:96750923_T > C) showed a relatively even distribution across the different cell types and clusters (Figure 5b). In contrast, the novel SNV positioned at 11:65440255_C > A in a non-coding exon of the gene *NEAT1* showed preferential expression in macrophage-like cells (Figure 5c). 

**Figure 6:**
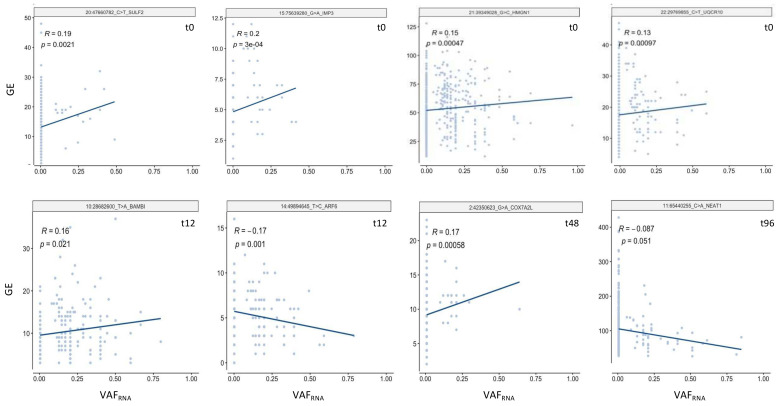
Examples of significant (FDR  =  0.05) cis-scReQTL correlations between sceSNVs and the expression of their harboring gene.

Finally, we assessed whether the expression of sceSNVs correlated with the expression of their harboring gene, for which we applied the linear regression model implemented in cis-scReQTL [11]. For this analysis, we used sceSNVs detected in five and more cells (between 35 and 70 sceSNVs per dataset). Across the four datasets, we identified a total of 20 cis-scReQTLs at a significance level of *p* < 0.05 (Figure 6 and Supplementary Figure S6). We indeed observed weak to moderate relationships, mostly due to the small number of cells expressing the variant SNV allele. This is expected for novel mutations, and also reflects the relatively small overall number of studied cells (1250–2887 cells per sample). We expect that by including a higher number of cells per sample, more recent and future scRNA-seq studies will enable improved correlation analyses.

## 4. Discussion

In this study, we performed an initial assessment of SNV calls from individual barcode-stratified scRNA-seq alignments. Our analysis shows that this strategy identifies a significantly higher number of SNVs as compared to variant calls on pooled scRNA-seq data. Specifically, even after high stringency filtering, in the individual cell alignments, we could identify at least a two-fold higher number of SNVs, as compared to the unfiltered union of SNVs called in the pooled scRNA-seq, exome, and genome sequencing data. Furthermore, we demonstrated that sceSNVs are substantially enriched in novel genetic variants and coding functional annotations. 

We found that SNVs called exclusively in the individual alignments—sceSNVs—possess several striking characteristics. First, sceSNVs are substantially enriched in previously undescribed variants. This finding is not surprising, as sceSNVs are seen in up to 5% of the cells in a dataset (most often in only one cell) and therefore likely to contain a high proportion of de novo SNVs. De novo SNVs can arise in most normal and tumor cells [33] but are only possible to be retained in the germline, in germline tissues. Therefore, fewer sceSNVs are currently reported in DbSNP, where the vast majority of SNVs are called from pooled germline DNA datasets. Hence, barcode-stratified SNV calls can facilitate studies on the occurrence and the evolution of de novo genetic variants. Most importantly, analyses like the one exemplified here can distinguish a setting to study newly occurring SNVs, thereby facilitating studies on both SNV occurrence and selection drivers.

We note that, while technical factors resulting in false-positive variant calls cannot be excluded, in this study we made every effort to minimize them. First, we used data generated on an UMI-utilizing scRNA-seq-based platform (10x Genomics), which is targeted to address the technical artifacts of PCR duplication. Second, for the individual alignment variant calls, we applied very stringent criteria for SNV filtering, based on quality and call confidence for both GATK and Strelka, followed by the removal of calls in difficult genomic regions (see Methods). Third, we visually examined (IGV) the alignments of over 200 sceSNV (Table 1) and, for all of them, we observed concordance with a high confidence call.

Therefore, in scRNA-seq data, we cannot exclude the possibility of an RNA-editing origin for some of the SNVs. However, we find the probability of RNA editing to be low since none of these loci were listed in RNA-editing databases; additionally, we removed from our analysis those repeated regions (Methods) that are known to harbor the vast majority of RNA-editing events.

Second, we find that the sceSNVs are significantly enriched in coding variants, especially in stop-codon and missense substitutions. This is likely to be related to the different rates of mutation generation, repair, and positive or negative selection. Many of the sceSNVs identified in the RNA of only a single cell are unlikely to be transmitted, including somatic functional sceSNVs that affect cell fitness and contribute to the cell fate, as well as neutral SNVs that follow passenger behavior. In addition, technical factors, including the 10x Genomics 3’UTR workflow, might play a role in differences in the observed SNV functional distributions. At this point, distinguishing biological from technical factors is challenging and requires a larger number of studies, including those focusing on mutation dynamics and evolution, and exploiting multiple heterogeneous sample sources. 

Third, we find that some sceSNVs might affect the expression of their harboring gene, thereby possibly exerting downstream effects. In this study, we find 20 significant cis-scReQTLs. This number is expected given the input size (up to 70 SNVs and up to 3000 cells per dataset), and, based on our previous studies, is likely to be significantly higher in larger datasets [11,18]. Furthermore, 10x Genomics 3’ scRNA-seq workflows naturally retain a high number of 3’-UTR-located variants (see Figure 3), which are acknowledged to exert regulatory effects on both gene expression and splicing [34,35,36]. Similarly, the substantial number of captured intronic SNVs can be utilized in the estimation of precursor and mature mRNA abundance [10]. Identifying cell-level SNVs and estimating their effects on the gene expression can help to define functionality for expression- and splicing-regulatory variants, as well as those variants potentially implicated in RNA velocity [37].

Regarding the data used, it is important to note that while we selected the MCF7 datasets due to their technical suitability, namely, matched scRNA-seq and DNA sequencing, the potential contribution of the bortezomib treatment to the transcriptional heterogeneity of an immortalized cell line is possible. Here we note that, as part of an ongoing related study on normal and tumor human tissues, we have observed a similarly higher rate of SNV discovery from the individual (vs. pooled) scRNA-seq alignments, as well as a higher proportion of novel SNVs (unpublished data).

## 5. Conclusions

Overall, our study indicates an immense potential for SNV assessment from individual cell scRNA-seq data. Given the growing accumulation of scRNA-seq datasets, cell-level variant assessments are likely to significantly contribute to our understanding of cellular heterogeneity and the relationship between genetics and functional phenotypes. It is of note that the approach used here, including barcode-stratified alignment generation and variant calls from the individual cell alignments, can be computationally expensive for scRNA-seq generated from a high number of cells. Therefore, methods for a cell-level variant call from scRNA-seq data are highly in demand. Such methods can be applied in studies on normal and diseased (especially cancerous) tissues, where they can help to elucidate not only the SNV occurrence rate but also variant evolution and functionality.

## Figures and Tables

**Table 1:** Number of SNVs identified in the MCF7 sequencing datasets, in the exonic regions of the genes included in the ProfileOncoPanel (POPv.3).

Sample and Sequencing Approach	SRA id	N Cells	N SNVs GATK	N SNVs Strelka2	Bulk/Pooled GATK and Strelka2 (Union)	Sc Alignments GATK and Strelka2 (Intersection)	N sceSNVs	N sceSNVs by Mutect2
TES (POPv.3)	SRR5945460	na	395	390	409	na	na	na
WGS	SRR5945478	na	25	322	322	na	na	na
scRNA-seq t0	SRR10018149	1749	256	2312	2800 *	149	73 (49%)	6 (8%)
scRNA-seq t12	SRR10018150	2778	347	3373	3882 *	101	61 (60%)	7 (11%)
scRNA-seq t48	SRR10018151	1891	218	2639	3132 *	79	38 (48%)	1 (3%)
scRNA-seq t96	SRR10018152	1250	126	1481	1996 *	86	45 (52%)	2 (4%)

* The numbers include the SNVs called in TES and WGS shown above.

**Table 2:** The number of SNVs transcriptome-wise, and percent cells with scSNVs.

Sample	Pooled scRNA-seq Alignments (GATK and Strelka2 Union)	N sceSNVs (GATK and Strelka2 Intersection)	N sceSNVs by Mutect2	Max Percent Cells with sceSNVs	N sceSNVs in 2 and More Cells
scRNA-seq t0	489,048	13,385	1310 (9.8%)	90/1749 (5.2%)	636 (4.8%)
scRNA-seq t12	524,598	9470	936 (9.9%)	44/2778 (1.6%)	472 (5%)
scRNA-seq t48	446,779	7131	560 (7.8%)	33/1891 (1.8%)	318 (4.5%)
scRNA-seq t96	335,839	10,794	856 (7.9%)	30/1250 (2.4%)	429 (4%)

**Table 3:** Distribution of functional annotations between sceSNVs and pSNVs (chi-square comparisons).

Function	t0		t12		t48		t96	
	sceSNVs	pSNVs	sceSNVs	pSNVs	sceSNVs	pSNVs	sceSNVs	pSNVs
3-prime-UTR	8075 (60%)	6062 (15%)	5785 (61%)	6340 (18%)	4109 (57%)	5618 (17%)	6562 (61%)	5272 (19%)
chi-square *p*-value	10,883 *p* < 10^−7^		6981 *p* < 10^−7^		5092 *p* < 10^−7^		6403 *p* < 10^−7^	
missense	2010 (15%)	1129 (3%)	1562 (17%)	1513 (4%)	1323 (18%)	1388 (4%)	1744 (16%)	915 (3%)
chi-square *p*-value	2808 *p* < 10^−7^		1726 *p* < 10^−7^		1854 *p* < 10^−7^		2008 *p* < 10^−7^	
intron	1609 (12%)	24,634 (60%)	870 (9%)	19,247 (55%)	667 (9%)	17,559 (54%)	1045 (10%)	14,351 (52%)
chi-square *p*-value	9236 *p* < 10^−7^		6261 *p* < 10^−7^		4749 *p* < 10^−7^		5694 *p* < 10^−7^	
synonymous	897 (7%)	1365 (3%)	758 (8%)	1844 (5%)	580 (8%)	1756 (5%)	793 (7%)	1213 (4%)
chi-square *p*-value	287 *p* < 10^−7^		102 *p* < 10^−7^		76 *p* < 10^−7^		140 *p* < 10^−7^	
intergenic	276 (2%)	6484 (16%)	182 (2%)	4680 (13%)	161 (2%)	4475 (14%)	236 (2%)	4941 (18%)
chi-square *p*-value	1751 *p* < 10^−7^		997 *p* < 10^−7^		755 *p* < 10^−7^		1624 *p* < 10^−7^	
non-coding exon	250 (1.9%)	414 (1%)	142 (1.5%)	357 (1%)	102 (1.4%)	331 (1%)	200 (1.9%)	421 (1.5%)
chi-square *p*-value	60 *p* < 10^−7^		15 *p* = 0.00007		9 *p* = 0.002		6 *p* = 0.02	
5-prime-UTR	152 (1.1%)	816 (2%)	78 (0.8%)	1027 (2.9%)	118 (1.6%)	1098 (3.4%)	80 (0.7%)	622 (2.2%)
chi-square *p*-value	41 *p* < 10^−7^		135 *p* < 10^−7^		58 *p* < 10^−7^		96 *p* < 10^−7^	
splice	16 (0.12%)	105 (0.26%)	15 (0.16%)	120 (0.34%)	9 (0.13%)	103 (0.32%)	18 (0.17%)	78 (0.28%)
chi-square *p*-value	8 *p* = 0.005		8 *p* = 0.006		7 *p* = 0.008		4 *p* = 0.04	
stop	100 (0.75%)	6 (0.01%)	78 (0.82%)	6 (0.02%)	62 (0.86%)	2 (0.01%)	116 (%1.07)	5 (0.02%)
chi-square *p*-value	275 *p* < 10^−7^		253 *p* < 10^−7^		264 *p* < 10^−7^		275 *p* < 10^−7^	

## Data Availability

All the data analyzed in this study are supplied with the Supplemental Material or available as indicated in the cited publications.

## Supplementary Materials

The following are available online at https://www.mdpi.com/article/10.3390/genes12101558/s1, Supplementary Figure S1: Cell Features Distribution and Filtering, Supplementary Figure S2: Cellcycle and batch effects removal, Supplementary Figure S3: Cell types, Supplementary Figure S4: Shared and exclusive SNVs, Supplementary Figure S5: Cell distribution of sceSNVs, Supplementary Figure S6: scReQTLs, Supplementary Table S1: ScSNVs four timepoints oncoPanel, Supplementary Table S2: ScSNVs four timepoints wholeTranscriptome.
