# Supplementary material for: Improved SNV Discovery in Barcode-Stratified scRNA-seq Alignments
Source: Genes (Basel). 2021 Sep 30;12(10):1558. doi: 10.3390/genes12101558 (PMC8535975; doi:10.3390/genes12101558)
Supplement: Supplementary file 1 [file genes-12-01558-s001.zip › Supplementary_Figures_092421/Supplementary_Figure 6_scReQTLs.pptx]

## Slide 1
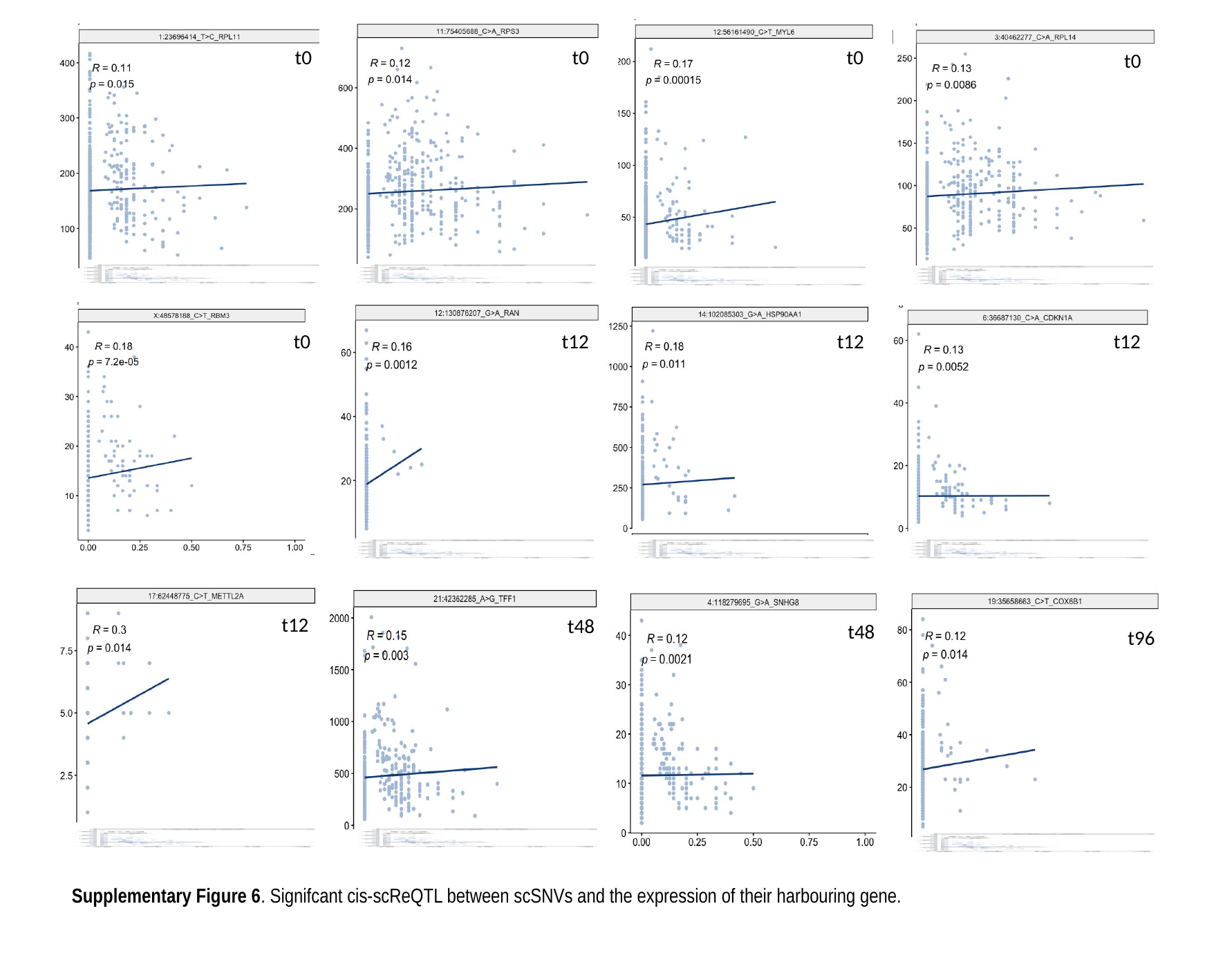

t0
t0
t0
t0
t0
t12
t12
t12
150
t12
t48
t48
t96
Supplementary Figure 6. Signifcant cis-scReQTL between scSNVs and the expression of their harbouring gene.
