# Supplementary material for: Improved SNV Discovery in Barcode-Stratified scRNA-seq Alignments
Source: Genes (Basel). 2021 Sep 30;12(10):1558. doi: 10.3390/genes12101558 (PMC8535975; doi:10.3390/genes12101558)
Supplement: Supplementary file 1 [file genes-12-01558-s001.zip › Supplementary_Figures_092421/Supplementary_Figure 5_Cell_distribution_of_sceSNVs.pptx]

## Slide 1
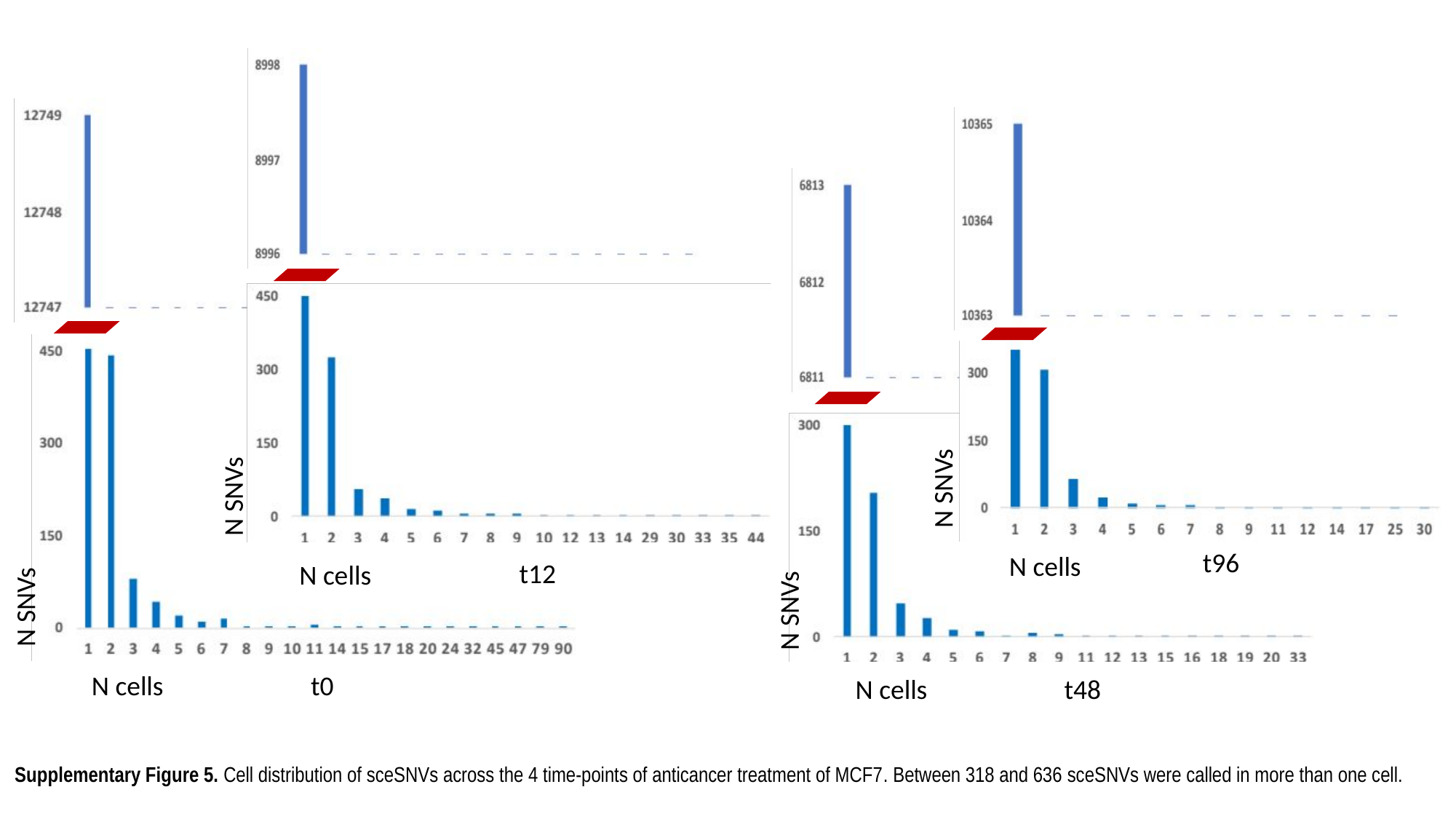

N SNVs
N SNVs
t96
N cells
t12
N cells
N SNVs
N SNVs
N cells
t0
t48
N cells
Supplementary Figure 5. Cell distribution of sceSNVs across the 4 time-points of anticancer treatment of MCF7. Between 318 and 636 sceSNVs were called in more than one cell.
