# Supplementary material for: Improved SNV Discovery in Barcode-Stratified scRNA-seq Alignments
Source: Genes (Basel). 2021 Sep 30;12(10):1558. doi: 10.3390/genes12101558 (PMC8535975; doi:10.3390/genes12101558)
Supplement: Supplementary file 1 [file genes-12-01558-s001.zip › Supplementary_Figures_092421/Supplementary_Figure 3_Cell_types.pptx]

## Slide 1
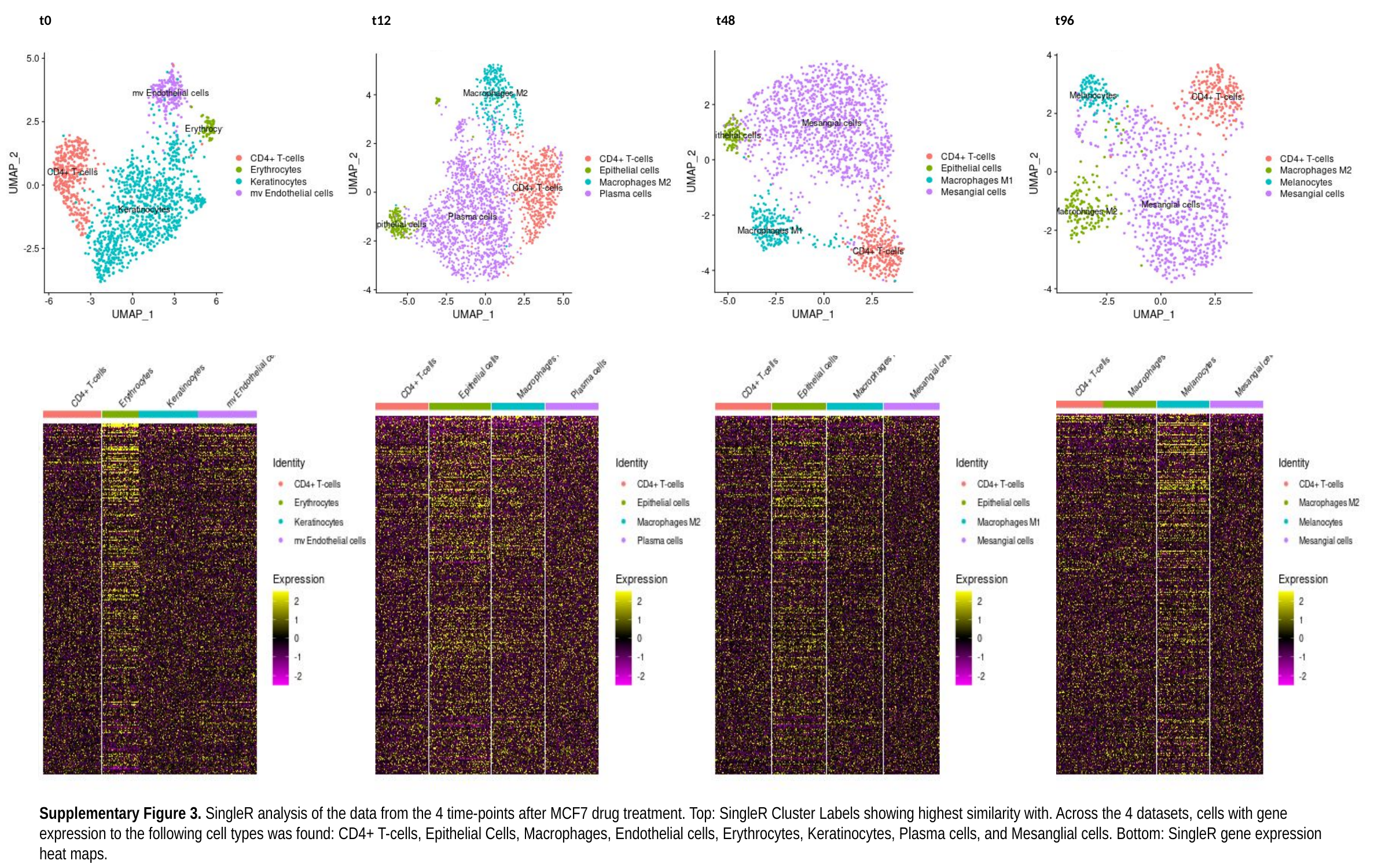

t0
t12
t48
t96
a
b
Supplementary Figure 3. SingleR analysis of the data from the 4 time-points after MCF7 drug treatment. Top: SingleR Cluster Labels showing highest similarity with. Across the 4 datasets, cells with gene expression to the following cell types was found: CD4+ T-cells, Epithelial Cells, Macrophages, Endothelial cells, Erythrocytes, Keratinocytes, Plasma cells, and Mesanglial cells. Bottom: SingleR gene expression heat maps.
