# Supplementary material for: Improved SNV Discovery in Barcode-Stratified scRNA-seq Alignments
Source: Genes (Basel). 2021 Sep 30;12(10):1558. doi: 10.3390/genes12101558 (PMC8535975; doi:10.3390/genes12101558)
Supplement: Supplementary file 1 [file genes-12-01558-s001.zip › Supplementary_Figures_092421/Supplementary_Figure 1_Cell_Features_Distribution and Filtering .pptx]

## Slide 1
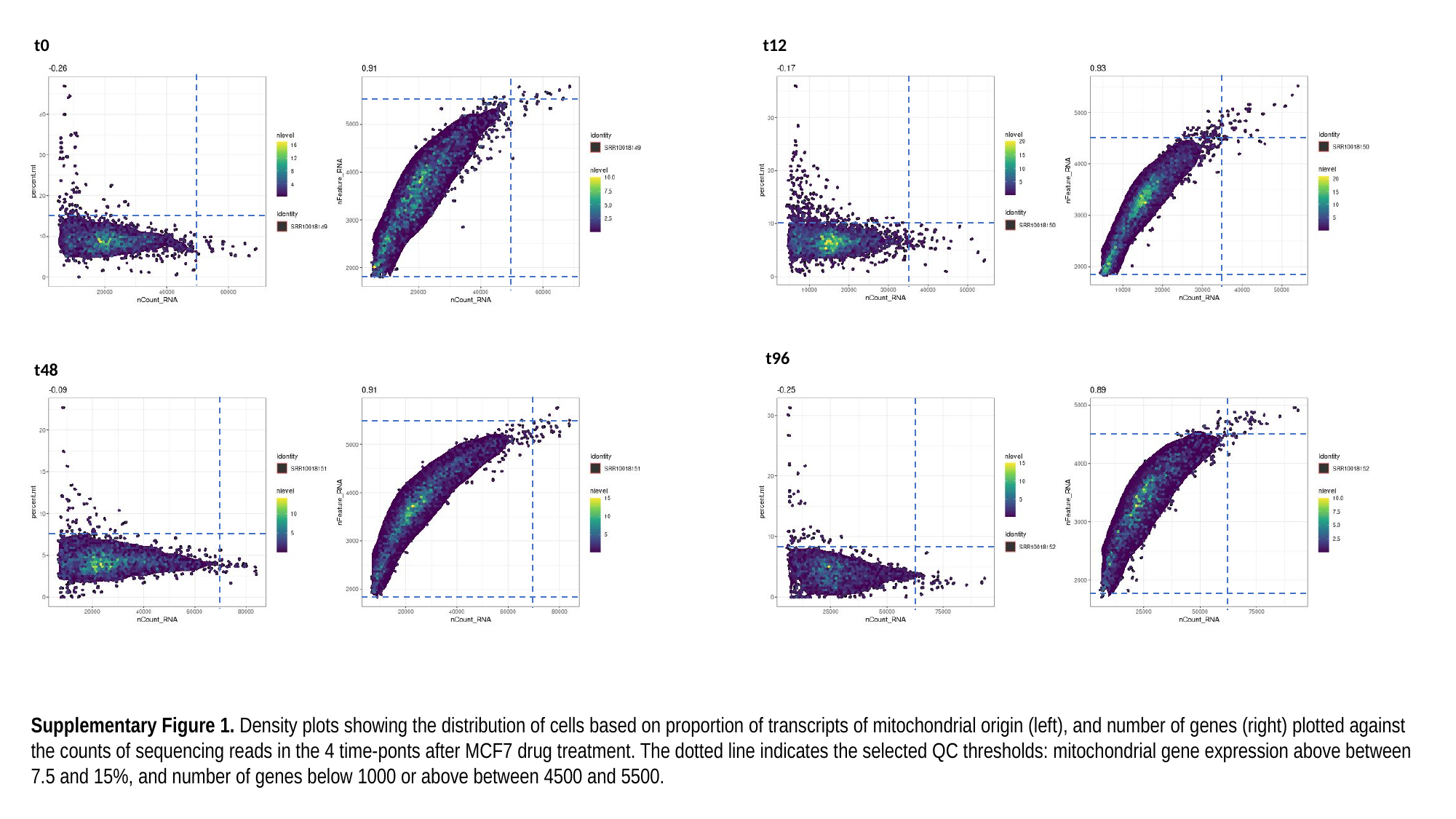

t0
t12
t96
t48
Supplementary Figure 1. Density plots showing the distribution of cells based on proportion of transcripts of mitochondrial origin (left), and number of genes (right) plotted against the counts of sequencing reads in the 4 time-ponts after MCF7 drug treatment. The dotted line indicates the selected QC thresholds: mitochondrial gene expression above between 7.5 and 15%, and number of genes below 1000 or above between 4500 and 5500.
