# Supplementary material for: Improved SNV Discovery in Barcode-Stratified scRNA-seq Alignments
Source: Genes (Basel). 2021 Sep 30;12(10):1558. doi: 10.3390/genes12101558 (PMC8535975; doi:10.3390/genes12101558)
Supplement: Supplementary file 1 [file genes-12-01558-s001.zip › Supplementary_Figures_092421/Supplementary_Figure 2_Cell_cycle_and_batch_effects_removal.pptx]

## Slide 1
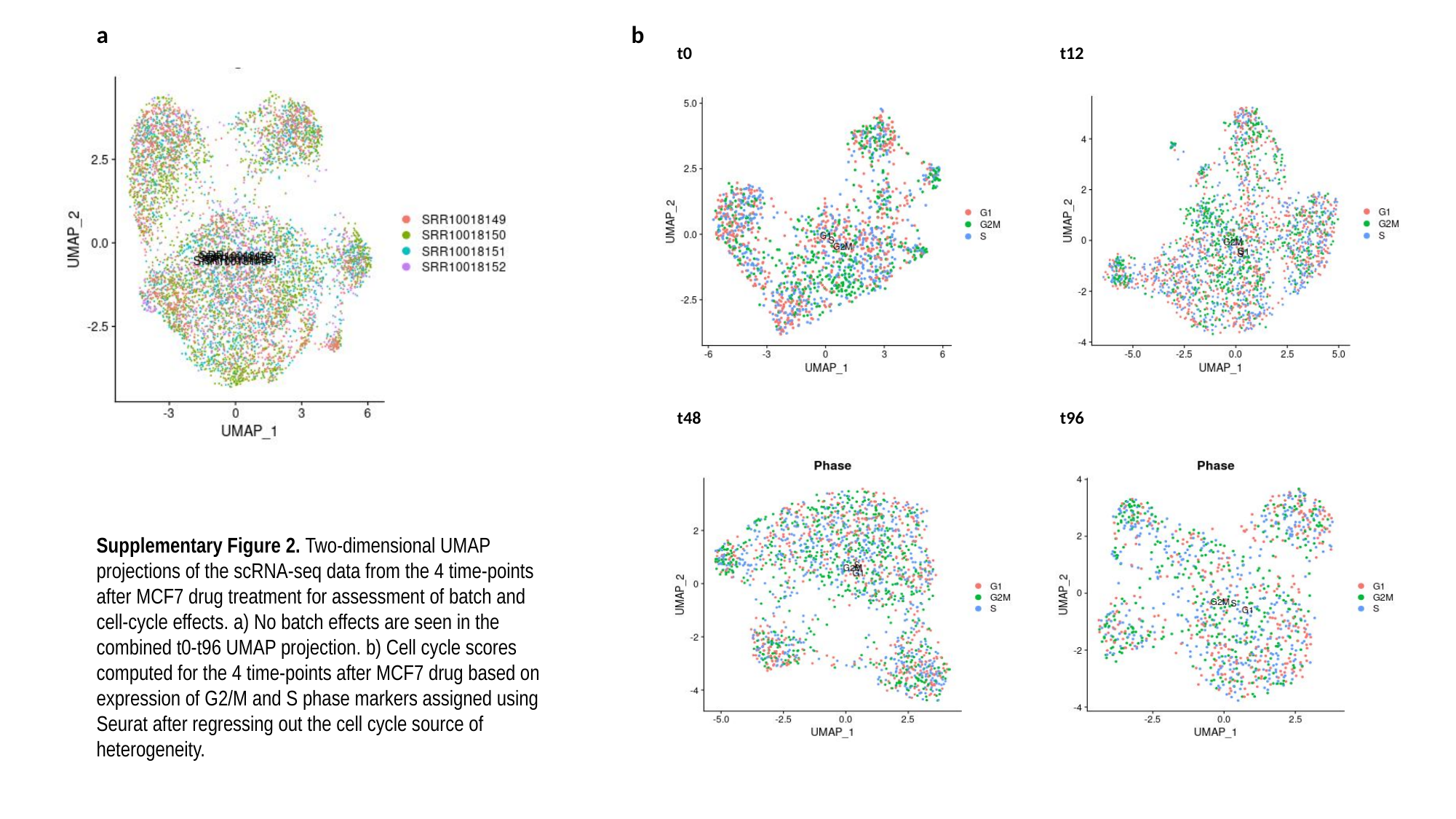

a
b
t0
t12
t48
t96
Supplementary Figure 2. Two-dimensional UMAP projections of the scRNA-seq data from the 4 time-points after MCF7 drug treatment for assessment of batch and cell-cycle effects. a) No batch effects are seen in the combined t0-t96 UMAP projection. b) Cell cycle scores computed for the 4 time-points after MCF7 drug based on expression of G2/M and S phase markers assigned using Seurat after regressing out the cell cycle source of heterogeneity.
